# Cards and the Doctor

**Published:** 1908-09-12

**Authors:** 


### The Practitioner's Relaxations and Hobbies.

### CARDS AND THE DOCTOR.

To the medical profession the "ancient and interesting game of cards" offers many attractions, even though its variations have become so numerous and so involved that the length of one man's life is insufficient thoroughly to become acquainted with them. In the first place the game is said to have been invented by the medical attendants of a mad king of France, and if we wish to make a list of the discoveries which the world owes to the energies of the profession, I should be tempted to include in it cards. But it is not solely because of its quasi medical origin that the game and its innumerable variations should prove of interest to the practitioner. There are other and greater reasons. And in setting them forth, let me state at once that I have no quarrel with those who regard the game as immoral. Everyone is justified in maintaining his own individual opinion, whether or not it agrees with those of the rest of his race, and if there are some of us who take it to be an offence against the better part of humanity to indulge in card-playing, I refrain from commenting on his reasoning, because I bear in mind that many members of the profession—now and in times past—have held extreme and eccentric views. The celebrated Messenger Monsey, for instance, thought it "ungodlike" to play bowls, and we know of one sane and respectable practitioner who deems it a crime scarcely more atrocious than a breach of professional etiquette to play golf. But these are extremes, and I do not for a moment wish to put them forward as examples. All I wish to state is that the knowledge that such people do exist in our liberal and open-minded profession makes me tolerant of those of my colleagues who have scruples about joining in a friendly game of Bridge.

As a relaxation for the medical man, cards, of all indoor games, stands easily first. The superfluities of chess, the intricacies of draughts, and the evanescent interest that halma arouses, pale before it, and the only indoor game that seriously rivals it, as a doctor's recreation, is the little known "Kriegspiel." The man who regards his cards as a serious matter, as a true relaxation, will never become a gambler at the game. Nor will he waste over it so much time, patience, and brain work as he will over chess or draughts. A knowledge of one or two card games, and an ability to play them with average skill—an ability which is, after all, easily acquired —will make him a social acquisition, and will undoubtedly tend to enhance his popularity. Nor need I point out the utility of the game as a mental and moral training. Patience, toleration, courtliness and courtesy, forbearance, self-restraint, determination, and will power—all these are cultivated by the true player at cards, and each and all of them are virtues which the practitioner would do well assiduously to cultivate. For these reasons, perhaps, it is that one finds cards generally popular with the members of the profession. All the great leaders of medicine during the last century were, almost without exception, expert card players, and not to be a good whist player was reckoned such a failing in a professional man during the eighteenth century that we find the biographer of one medical eminency apologetically remarking that his hero "unfortunately dislikes cards and could never be persuaded to take a hand at a party, for which reason he lost many of his more fashionable patients." This gentleman, it is interesting to note, was not the scornful and unconventional Abernethy, nor yet the outspoken Radcliffe, whom neither King William III.'s three kingdoms nor his fame could persuade to have his Majesty's legs; but a much more staid and worthy, though equally eminent, physician.

Of the many variations that compete for the medical man's consideration, there are some that stand out above all the rest as eminently worthy of closer acquaintance. We have heard one eminent London consultant declare that "every medical man ought to know how to play poker," and for a specialist, at least, a knowledge of the wiles of that game ought to come in very useful sometimes. In all seriousness, however, the game of poker, now so rarely played in the family circle, is one of the finest of card games, demanding as it does patience and pertinacity, a strict control over the feelings, and an unlimited fund of good-natured toleration. In all card games an element of luck must necessarily enter, and in some, poker among others, that element is necessarily larger than in others. But there are few indoor games which depend on pure skill, and those few are, because of their intricacy and the amount of mental exertion which they demand, hardly to be classed as pure relaxations. To some, it is true, a hard fought game of chequers is a true relaxation, and in so far as all change of occupation is of necessity a mental rest, it is conceivable that an involved "end game" of chess is an equally true relaxation; but on the whole few practitioners have the capacity, after a hard day's work, to indulge in such severe mental strain as the correct playing of these games demands. Cards, on the contrary, needs far less mental application, and offers much greater relaxation, and as such it is permissible to claim for it the merit of being an almost ideal game for the doctor. The severely correct style of card-playing, immortalised in the celebrated Mrs. Battle, is getting out of fashion, and as a relaxation most people prefer Lamb's way of playing—as a pastime, not as an abstruse science.

It is in many ways a pity that Bridge has displaced the older, and, to the writer at least, equally fascinating game of whist. But it is with us; and the practitioner who wishes to be perfect should have an intimate knowledge of the game, since it is the most popular of all card games. As a game it is perhaps far inferior to the popular German card game "skat," of which it is a weak variation; but life is too short for most of us to master the whole art and practice of skat-playing. The correct whist player will have little difficulty in mastering the essentials of Bridge. There are several excellent manuals of the game on the market, and a few lessons will clear up practical difficulties and lay a solid foundation for future play. Instinct and intuition are, perhaps more necessary at Bridge play than in any other game of cards, with the exception of poker. The declaration especially demands experience and a fine perception of negative and positive possibilities. Euchre, baccarat, and solo have all their praiseworthy points, and they have this much in common, namely that they demand from the player a certain amount of skill and attention, and a subtle recognition of his own and his partner's strength and weaknesses. For, taken in the gross, card-playing is an admirable school for the study of human nature, and as such alone it should have the attention of the medical man.

The card-player has an admirable hobby at hand if he is blessed (or cursed) with that magpie propensity, the collecting habit. The collection of cards, playing-cards, is one of the most fascinating hobbies that I know of. It is indeed not an easy one. To obtain, for instance, examples of historic playing cards is usually very difficult, and entails an expenditure of money and time, which few men in active practice can afford. I know, however, of one colleague who, by dint of perseverance and patience, has amassed a superb collection of playing-cards, in which every country, every race almost, is represented. His collection, which is kept in a large folio album, suitably bound, contains some famous examples. The packs are inserted in the manner of post-cards, wherever possible all the fifty-two cards of a pack being included, and the array of colour and the combination of design are exceptionally interesting and beautiful. The collection has taken him almost a lifetime to obtain, and many of his choicest specimens are gifts from poor patients. One of such is a pack perforated by a bullet, a memento of the Crimean War; another is a mutilated pack which accompanied Emin Pasha across the African continent; while a third, perhaps the most humanly interesting of all, is a makeshift pack, manufactured out of a table cloth and the red shirt of a Garibaldian.

